# Supplementary material for: Current sequence-based models capture gene expression determinants in promoters but mostly ignore distal enhancers
Source: Genome Biol. 2023 Mar 27;24:56. doi: 10.1186/s13059-023-02899-9 (PMC10045630; doi:10.1186/s13059-023-02899-9)
Supplement: Supplementary file 2 — Additional file 2. PDF containing methods, figures and analysis applicable to the manual matching of CAGE tracks to GTEx tissues. [file 13059_2023_2899_MOESM2_ESM.pdf]

Manual matching of CAGE tracks to tissues

This supplementary file reports on the ability of Enformer and Basenji2 to predict gene expression in human tissues when we manually match CAGE tracks to the respective tissues and stages of development.

Matching for GTEx tissues

To match CAGE tracks to GTEx tissues, we used the following keyword searches:

Adipose - Subcutaneous : 'Adipocyte - subcutaneous'
Adipose - Visceral (Omentum) : 'Adipocyte - omental'
Adrenal Gland : 'adrenal gland'
Artery - Aorta : 'aorta'
Artery - Coronary : 'Coronary Artery'
Artery - Tibial : 'artery, adult'
Bladder : ':bladder, adult'
Brain - Amygdala : 'amygdala'
Brain - Anterior cingulate cortex (BA24) : 'brain,', 'cortex'
Brain - Caudate (basal ganglia) : 'caudate'
Brain - Cerebellar Hemisphere : 'cerebellum'
Brain - Cerebellum : 'cerebellum'
Brain - Cortex : 'brain,', 'cortex'
Brain - Frontal Cortex (BA9) : 'front'
Brain - Hippocampus : 'hippo'
Brain - Hypothalamus : 'thalamus'
Brain - Nucleus accumbens (basal ganglia) : 'accumbens'
Brain - Putamen (basal ganglia) : 'putamen'
Brain - Spinal cord (cervical c-1) : 'spinal cord'
Brain - Substantia nigra : 'nigra'
Breast - Mammary Tissue : 'mamma', 'breast,'
Cells - Cultured fibroblasts : 'fibro'
Cells - EBV-transformed lymphocytes : 'lymphocyte', 'Natural Killer', '\+ T cell', '\+ B cell'
Cervix - Ectocervix : 'cervix'
Cervix - Endocervix : 'cervix'
Colon - Sigmoid : 'colon,'
Colon - Transverse : 'colon,'
Esophagus - Gastroesophageal Junction : 'esoph'
Esophagus - Mucosa : 'esoph'
Esophagus - Muscularis : 'esoph'
Fallopian Tube : 'uterus','uterine'
Heart - Atrial Appendage : 'heart'
Heart - Left Ventricle : 'ventricle'
Kidney - Cortex : 'kidney,'
Kidney - Medulla : 'kidney,'

Liver : 'liver,'
Lung : 'lung,'
Minor Salivary Gland : 'salivary gland'
Muscle - Skeletal : 'skeletal muscle'
Nerve - Tibial : 'nerve'
Ovary : 'ovary, a'
Pancreas : 'pancreas,'
Pituitary : 'pitu'
Prostate : 'prostate, a'
Skin - Not Sun Exposed (Suprapubic) : 'skin,'
Skin - Sun Exposed (Lower leg) : 'skin,','palm'
Small Intestine - Terminal Ileum : 'small intestine'
Spleen : 'spleen'
Stomach : 'stomach'
Testis : 'testis'
Thyroid : 'thyroid,'
Uterus : 'uterus'
Vagina : 'vagina'
Whole Blood : 'blood,','Whole blood'

If the keyword search returns more than one match, we computed the average across the resulting tracks.

Matching for Cardoso-Moreira et al.

To match CAGE tracks to tissues, we used the following keyword searches:

Brain: ‘brain,’
Cerebellum: ‘cerebellum’
Heart: ‘heart,’
Kidney: ‘kidney,’
Liver: ‘liver,’
Ovary: ‘ovary,’
Testis: ‘testis,’

To match development stages, we used the following procedure. If, for a particular track, there was a ‘fetal’ or ‘newborn’ version, then we used this track for all pre-birth stages of development, as well as ‘newborn’, ‘infant’ and ‘toddler’. For the heart tissue, some CAGE tracks were annotated as diseased. We excluded those. For ovary and testis, only CAGE tracks annotated as ‘adult’ were available.

Results

Unsurprisingly, the manual matching generally leads to less predictive power than what can be achieved with the ridge regression. On the GTEx between-gene task, we observe a drop in performance of c. 10% for both Basenji2 and Enformer (Fig X1). The effect on the between-condition task is more pronounced, as the correlation between predictions and measurements drops from r = 0.64 to r = 0.42 (Fig X2, Basenji2: r = 0.54 to r = 0.34). This is not surprising as the individual errors which arise from a tissue being matched badly compound once we study inter-tissue variation. In either case, Enformer clearly still outperforms Basenji2 by a large margin.

The trends we observe in the data are overall similar. Performance is once again uneven across tissues (Fig X3), but the ordering of tissues is mostly unchanged (rho = 0.75). Moreover, on the Cardoso-Moreira et al. dataset, we again observe that later stages of development are predicted less-well than earlier ones (Fig X4). Interestingly, rather than a more continuous decline, we now see a sharp drop in performance after birth. This may be an artifact of the fact that we use a hard cutoff for when to use fetal and when to use adult CAGE tracks, whereas the linear model can interpolate continuously.

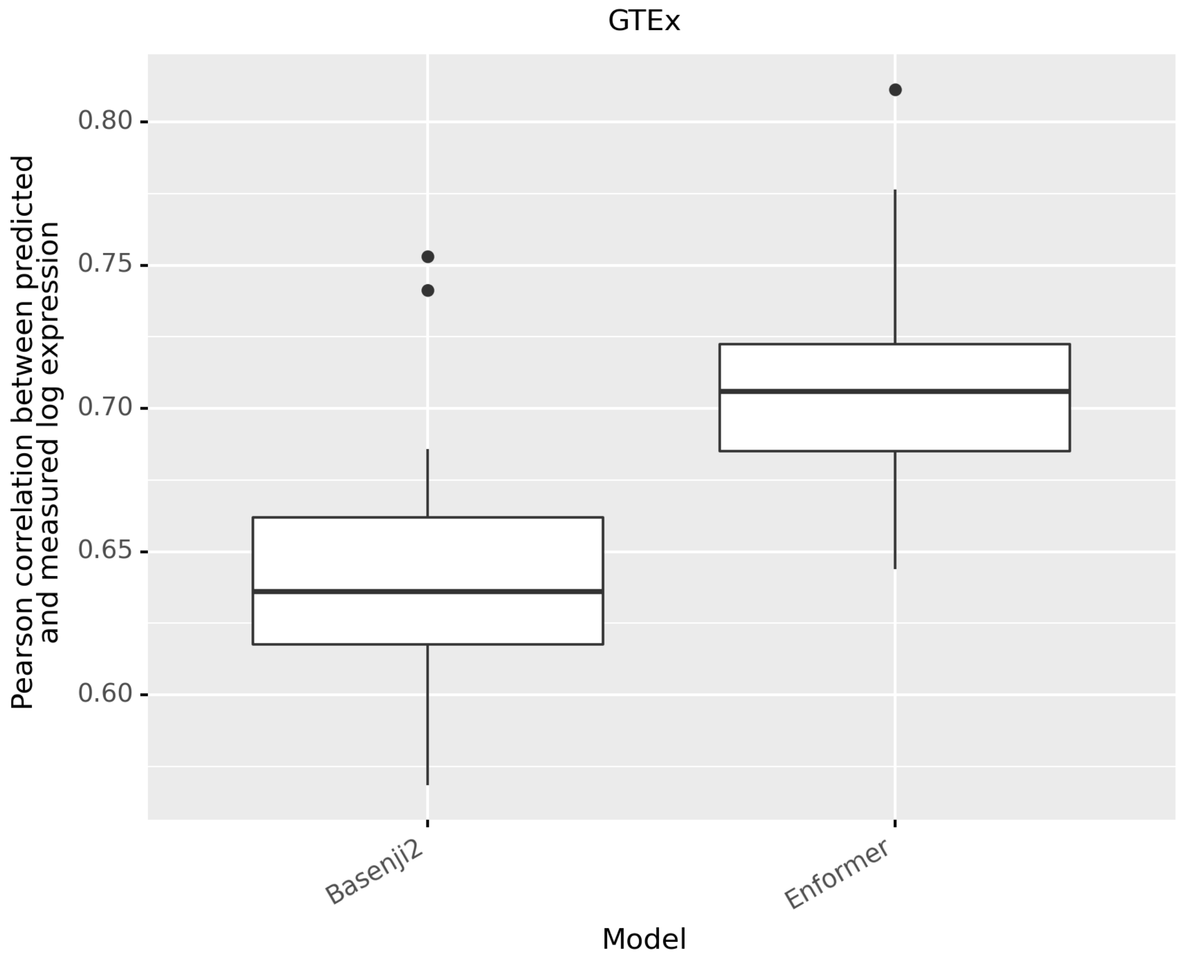

Figure X1: *Pearson correlation between predicted and measured log-transformed expression on GTEx tissues for different models, when using the manual matching. Compared to Fig 2A in the main text, the performance of Enformer for the median tissue drops from r = 0.79 to r = 0.71. However, the performance of Basenji2 drops to a similar extent (from r = 0.73 to r = 0.64).*

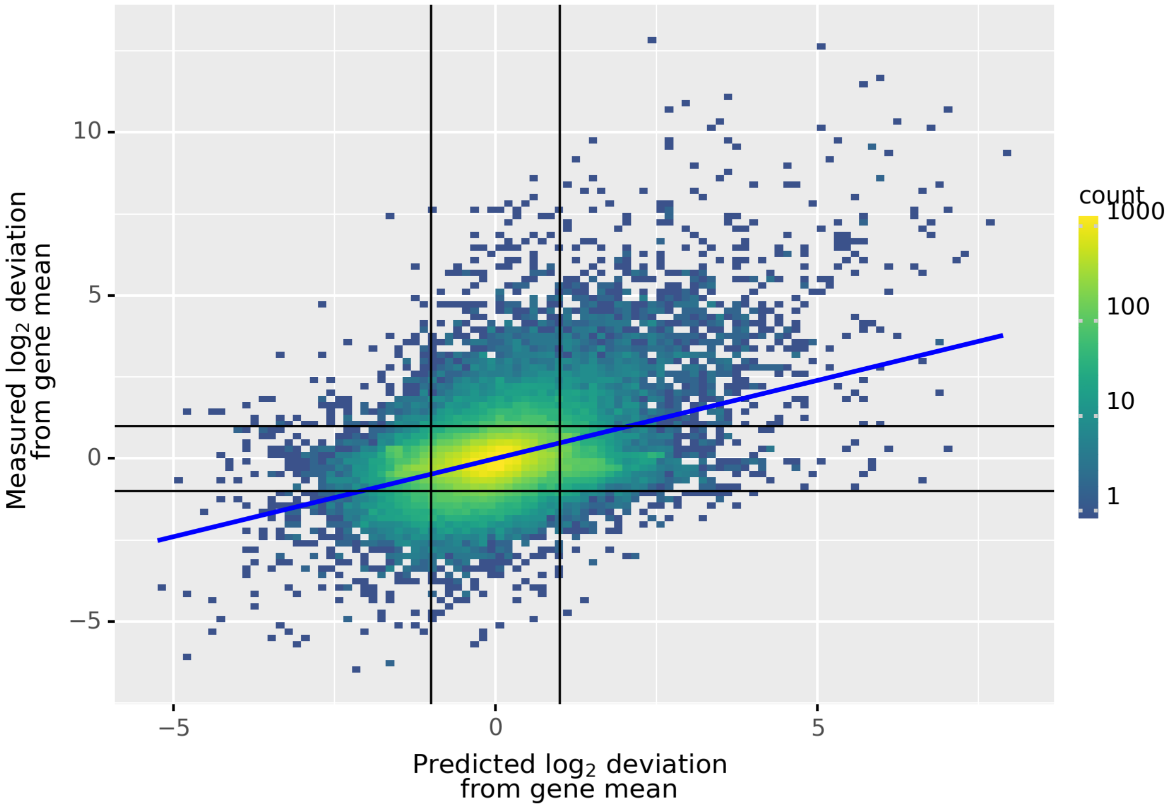

Figure X2: *Measured between-tissue deviations of gene expression against prediction. Compared to Fig 2D in the main text, the precision at 30% recall drops from 66% to 34%.*

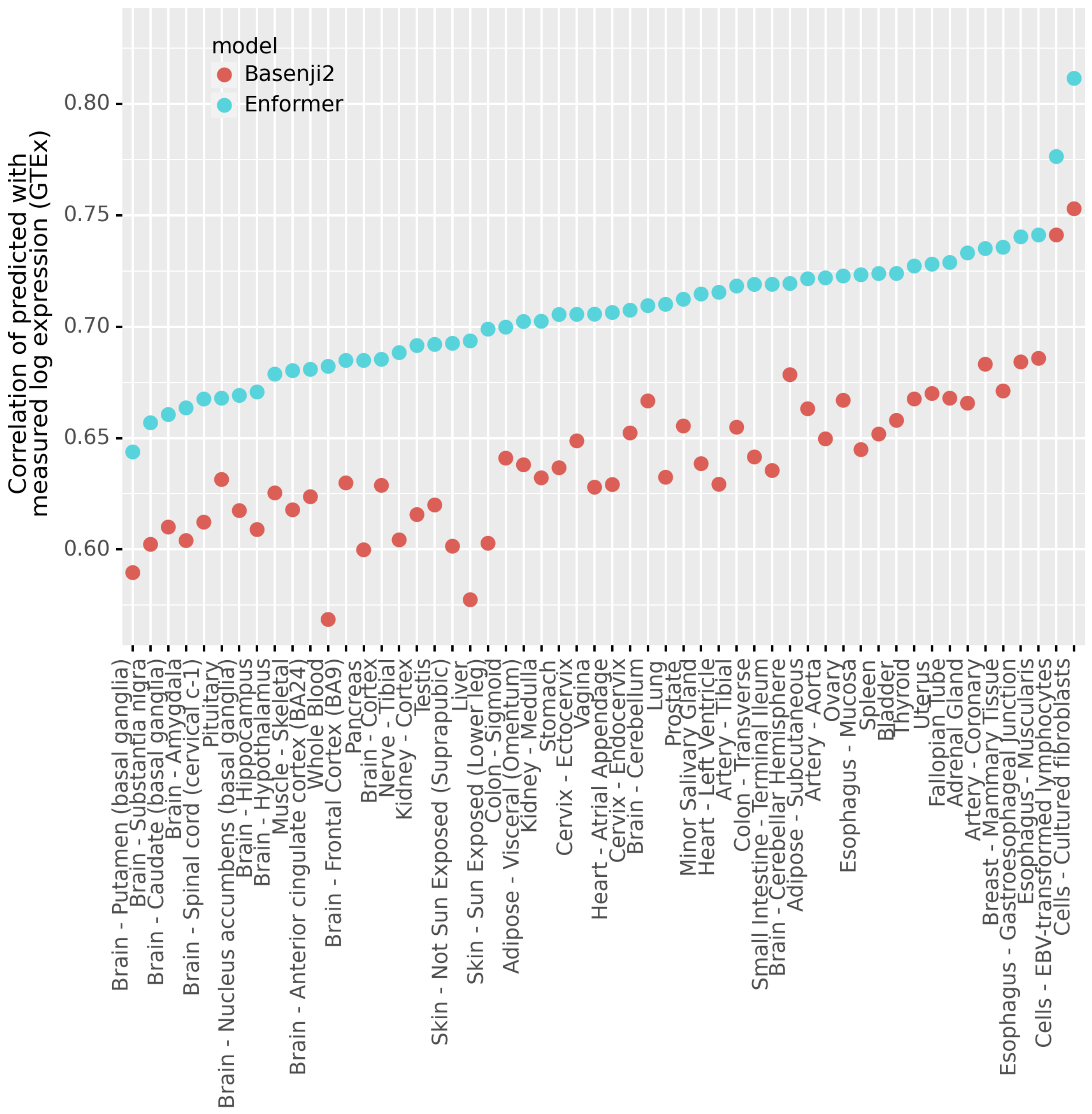

Figure X3: *Pearson correlation of predicted (using Enformer and Basenji2) and measured log expression for different GTEx tissues. We see that both models perform best for cultured cell-lines. Performance is lowest for brain tissues, pituitary and pancreas. If we compare the ranking implied by this figure with the one in Fig S1, we find a spearman correlation of rho = 0.75.*

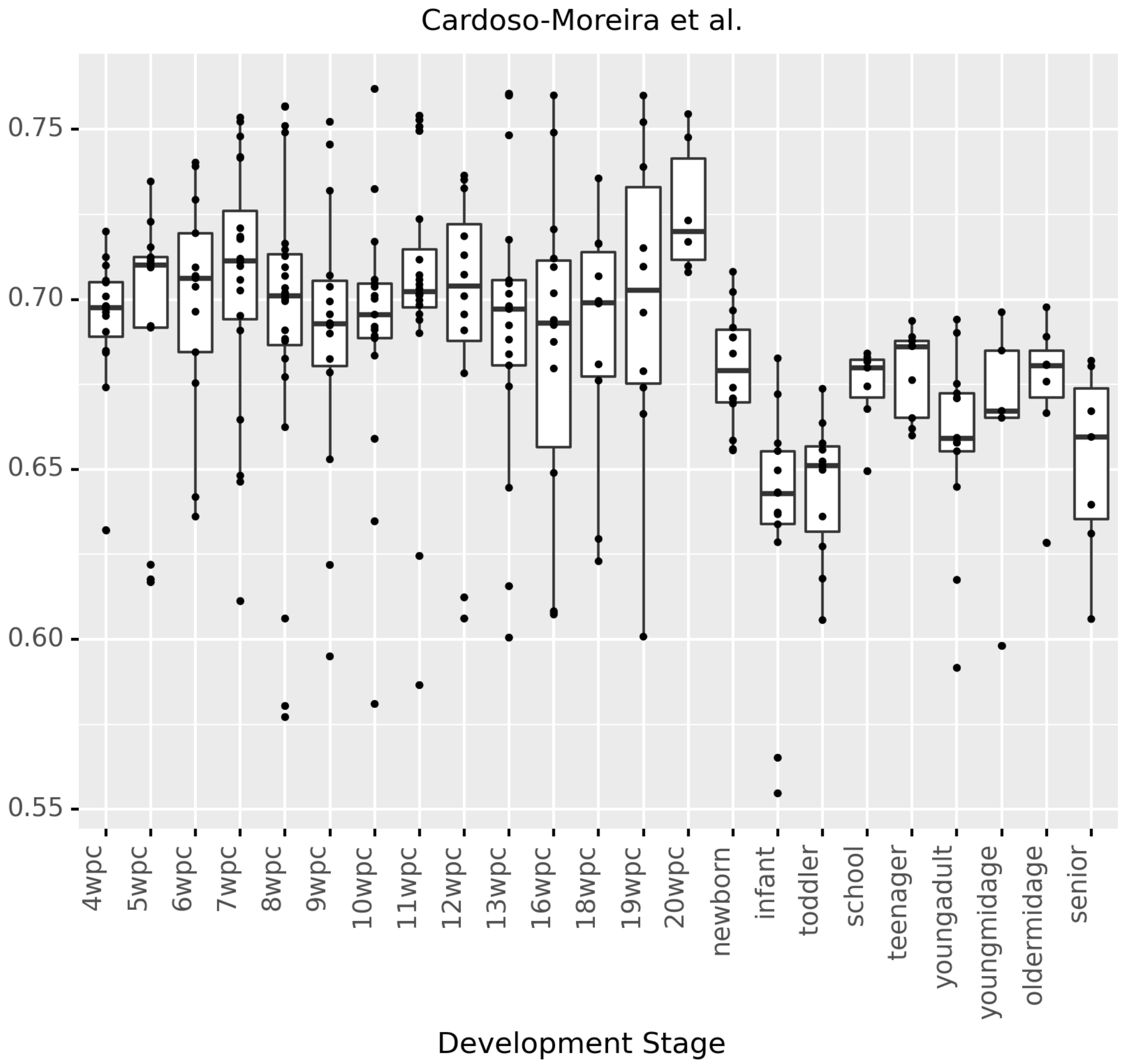

Figure X4: *Enformer predictions on developmental samples (Cardoso-Moreira et al. dataset). Compared to figure 2B in the main text, we see overall worse correlations but a similar trend, namely that Enformer predicts less-well for later stages of development.*
