# Supplementary material for: Current sequence-based models capture gene expression determinants in promoters but mostly ignore distal enhancers
Source: Genome Biol. 2023 Mar 27;24:56. doi: 10.1186/s13059-023-02899-9 (PMC10045630; doi:10.1186/s13059-023-02899-9)
Supplement: Supplementary file 3 — Additional file 3. Review history. [file 13059_2023_2899_MOESM3_ESM.docx]

Review History

**First round of review**

**Reviewer 1**

**Were you able to assess all statistics in the manuscript, including the appropriateness of statistical tests used?**

Yes, and I have assessed the statistics in my report.

**Comments to author:**

Were you able to assess all statistics in the manuscript, including the appropriateness of statistical tests used?

Karollus, et al. present a careful and thorough evaluation of large-scale sequence-based neural networks for representing gene regulatory activities. The study focuses on the abilities of several state-of-the-art models, including Enformer, Basenji, and Xpresso, to represent the contributions of promoter and enhancer elements to gene expression. Performance of the various methods is carefully assessed using a wide range of published data that characterize various aspects of enhancer or promoter activities or the effects of sequence perturbations in the same. Of the studied methods, most attention is given to Enformer, a recent model that uses a very large receptive field (196kbp) when predicting regulatory activities. The study validates the ability of Enformer to accurately predict gene expression in human tissues, and further demonstrates that both Enformer and Basenji provide accurate predictions of promoter expression output. However, the study also convincingly demonstrates that, despite its large receptive field, Enformer derives most of its predictions from sequence that is within ~30kbp of a gene's TSS. While Enformer can still prioritize/rank long-range enhancer-promoter interactions with reasonable accuracy, it systematically under-estimates the effect sizes of long-range enhancer contributions to expression.

This is a very valuable study to the field, as it not only provides a thorough independent evaluation of state-of-the-art neural networks for gene regulation, but also identifies a critical shortcoming in the current approaches. I have few comments on the manuscript; the evaluations appear appropriate and careful, and the manuscript is very well written.

The Discussion section contains useful commentary on the findings and challenges for the future. In it, the authors hypothesize that escalating class imbalance is the reason that current models fail to capture the effect of long-distance enhancers. This is a reasonable explanation, but I wondered if it could be tested using in silico sequences? Could validated distal enhancers be varied (in silico) in their positions w.r.t. the TSS to demonstrate that distance is a strong determinant of predicted effect size? In other words, how far do you need to "move" an enhancer before Enformer predicts the correct effect size? And does Enformer then over-predict effect size if you move the enhancer too close to the TSS?

The Discussion also suggests that predicting how distal enhancers are linked to target genes may be required for models to learn long-range regulatory interactions. I noted that Supplementary Figure 12 contains one approach ("Enformer-ABC") that may be relevant to this discussion. While I recognize that evaluating the Enformer-ABC model is outside of the scope of the current study, it may be of interest to mention the possibilities of this approach in the relevant section of the Discussion.

**Reviewer 2**

**Were you able to assess all statistics in the manuscript, including the appropriateness of statistical tests used?**

Yes, and I have assessed the statistics in my report.

**Comments to author:**

In this work, Karollus et al. undertake a deep investigation of state-of-the-art models for predicting expression from sequence. Such an investigation is very timely as several deep learning approaches have received wide attention in recent years and each claims to be highly accurate in predicting gene expression, but the concept of "predicting expression" is rather nuanced and multi-faceted and it is not quite clear what exactly these methods are good at and what their weaknesses might be. The authors test the leading methods for this task in a wide variety of settings and make clear-cut inferences. The evaluation procedures are designed with care and a lot of effort. The work is impressive in its examination of a number of different sequence-expression datasets and reaching coherent conclusions from a harmonized modeling of these diverse sources. The analysis reported in Figure 3, showing the interplay of distance from TSS, cis-regulatory signal and model performance, is very nicely done and presented. I believe this in itself will be of great value to readers. The analysis reported in Figure 4 presents useful insights into the capabilities of the Enformer model. The in silico experiment suggesting that disruption of a CAT-box may be responsible for a particular observation related to placement of the TATA box is appealing. Through Figure 5, the authors offer convincing evidence that even the best sequence models today are poor at predicting regulatory impact of distal enhancers and distal eQTLs. This is a very important message for the community, and in my opinion the demonstration is sound and clear.

All in all, this is an excellent and highly significant work. I have only a couple of minor suggestions for improvement of the manuscript.

Minor comments:

The first subsection of Results reads a little abrupt and hurried as it stands. It seems to assume that the reader has already read the Methods or is familiar with how the general paradigm of training and testing these models works.

The strategy of reporting accuracy on human tissues by applying a separately trained regressor (Figure 2B) appears somewhat misleading about predictive ability of Enformer, which only predicts the ENCODE CAGE tracks and the reported accuracy (and trends therein) may also reflect performance of the regressor (or some interaction between the Enformer predictions and the regressor). The authors should address this potential doubt that may arise in the reader's mind.

The concept of "reproducing an experiment in silico" should be gently introduced at the beginning of the Results section; it was not clear what exactly was meant by this phrase, though it did become clear during the course of reading the results section.

Line 130: Typo: should be lowercase 'w' in 'We'.

**Authors Response**

**Point-by-point responses to the reviewers’ comments:**

Reviewer #1:

Karollus, et al. present a careful and thorough evaluation of large-scale sequence- based neural networks for representing gene regulatory activities. The study focuses on the abilities of several state-of-the-art models, including Enformer, Basenji, and Xpresso, to represent the contributions of promoter and enhancer elements to gene expression. Performance of the various methods is carefully assessed using a wide range of published data that characterize various aspects of enhancer or promoter activities or the effects of sequence perturbations in the same. Of the studied methods, most attention is given to Enformer, a recent model that uses a very large receptive field (196kbp) when predicting regulatory activities. The study validates the ability of Enformer to accurately predict gene expression in human tissues, and further demonstrates that both Enformer and Basenji provide accurate predictions of promoter expression output. However, the study also convincingly demonstrates that, despite its large receptive field, Enformer derives most of its predictions from sequence that is within ~30kbp of a gene's TSS. While Enformer can still prioritize/rank long-range enhancer-promoter interactions with reasonable accuracy, it systematically under- estimates the effect sizes of long-range enhancer contributions to expression.

This is a very valuable study to the field, as it not only provides a thorough independent evaluation of state-of-the-art neural networks for gene regulation, but also identifies a critical shortcoming in the current approaches. I have few comments on the manuscript; the evaluations appear appropriate and careful, and the manuscript is very well written.

*>We thank the reviewer for their kind words.*

The Discussion section contains useful commentary on the findings and challenges for the future. In it, the authors hypothesize that escalating class imbalance is the reason that current models fail to capture the effect of long-distance enhancers. This is a reasonable explanation, but I wondered if it could be tested using in silico sequences? Could validated distal enhancers be varied (in silico) in their positions w.r.t. the TSS to demonstrate that distance is a strong determinant of predicted effect size? In other words, how far do you need to "move" an enhancer before Enformer predicts the correct effect size? And does Enformer then over-predict effect size if you move the enhancer too close to the TSS?

*>We implemented the analysis suggested by the reviewer and indeed observed a strong effect of distance. If shifted to the outer edge of Enformer‘s receptive field, the median validated enhancer will have a predicted effect of about 1% change in expression. This increases to 10% if brought within 10kb of the TSS and 16% if shifted to be 3kb from the TSS.*

*>The detection threshold in the CRISPRi experiments we analyzed seems to be around 10%. We in-silico shifted 505 validated enhancers, all of which are natively located 3kb away or further from the TSS. Of these, only 111 (~22%) had a predicted effect of 10% or larger at their native location, consistent with underestimation of the effects at large distance. By shifting those enhancers closer to the TSS in silico, we found 401 enhancers (~78%) predicted to have an effect larger than 10%.*

*>In regards to whether we can make the model to „over-predict“, we observe that there are 34 enhancers (~7%) which initially exhibit a predicted effect that exceeds the measured one. By shifting enhancers close to the TSS, this number increases to 221 (~44%). Thus, we can frequently make Enformer predictions reach and exceed the measured effect size, but not as a general rule. In median, the highest predicted effect size we can achieve through shifting is 1.4 percentage points below the measured one, which is reasonably close.*

*>In sum, we find that distance is a strong determinant of predicted effect size.*

*>However, this result must be interpreted with caution. When we shift a given enhancer, we are asking the model to predict the impact of distance all else equal. For this scenario, there actually exists experimental data that suggests what generally will happen in-vivo. Specifically, Zuin et al. used a transposon-based assay to vary the position of an enhancer relative to a fixed promoter in an otherwise unstructured region of DNA*. They found that the effect of the enhancer decays proportionally with distance in a way that is qualitatively very similar to what we see in the in-silico enhancer shift. So, in this sense, Enformer is probably correct to regard distance as a major determinant of effect size all else equal.*

**Zuin, Jessica, Gregory Roth, Yinxiu Zhan, Julie Cramard, Josef Redolfi, Ewa Piskadlo, Pia Mach, et al. 2022. “Nonlinear Control of Transcription through Enhancer–promoter Interactions.” Nature.* [*https://doi.org/10.1038/s41586-022-04570-y*](https://doi.org/10.1038/s41586-022-04570-y)*.*

*>However, in the CRISPRi studies, which lack this all else equal property as they measure a number of different enhancers in their respective native contexts, we observe a small subset of enhancers that exhibit large effects despite being far from their target TSS. In other words, it appears that the enhancer and/or the sequence context can heavily modulate the impact of distance.*

*>In this sense, what appears to be occurring is that Enformer has learned a distance relationship similar to what was identified in the transposon study, but inappropriately applies this in a more-or-less global manner, rather than in a more enhancer-specific fashion. We hypothesize that this is due to the escalating class imbalance. However, we think that the in-silico enhancer shift does not really provide much evidence in favor - or against - the hypothesis, for the reasons discussed above.*

*>We added the following section to the main text to (briefly) summarize the results of the enhancer shift and to discuss them in the context of the CRISPRi results:*

*>>We performed another in-silico experiment to study the impact of distance on enhancer strength. Specifically, we shifted validated enhancers, in their endogenous context, and measured the impact on expression at the TSS. In general we observed a strong effect of distance (Fig S23). If shifted to the outer edge of Enformer‘s receptive field, the median validated enhancer will have a predicted effect of about 1%. This increases to 10% if brought within 10kb of the TSS and 16% if shifted to be 3kb from the TSS. These observations are qualitatively similar to what was reported in a recent experimental study, which used a transposon system to vary the position of an enhancer relative to a fixed promoter in an otherwise unstructured region of DNA (Zuin et al. 2022).*

*>>It is important to note that this in-silico analysis and the experimental transposon study isolate the effect of distance for an enhancer all else equal whereas the CRISPRi studies compare the effect of different enhancers whose distance to the TSS is the result of evolution and not controlled by the experimenter. In this sense, they are not directly comparable. However, if we consider the two analyses together, we find that Enformer is probably correct that, absent other intervening factors, there exists a negative relationship between distance and the effect of an enhancer, but it improperly applies this decay in a more-or-less global manner, whereas in-vivo the impact of distance appears to be significantly more heterogeneous and modulated by other factors.*

*>On another note, the enhancer shift assay proposed by the reviewer does allow us to ask a related question: What would happen if we prevent Enformer from using distance as a determinant of enhancer strength? We can do this by simply shifting all enhancers to be equidistant to the TSS. We chose 20kb, as this is the median distance of CRISPRi validated enhancers. >If we focus only on CRISPRi validated enhancers, the ability of the model to correctly rank these in terms of their effect size actually slightly improves when we shift all enhancers to be equidistant (rho = 0.37 vs. rho = 0.31). This is not so surprising given Fig 5E, where we see that distance is not really predictive of the CRISPRi measured effect of validated enhancers.*

*>However, distance is very useful to distinguish validated from non-validated enhancers. Specifically, the ability of Enformer to distinguish validated and non- validated enhancers is almost halved when we equalize all distances: The average precision drops from 0.17 to 0.09, which is only slightly above the random classifier’s performance of 0.07. >We think this illustrates the difficulty that the escalating class imbalance represents. An ideal model of gene expression would be capable of determining how distance impacts each enhancer individually. However, if a model has sufficient difficulties with this - as seems to be the case for Enformer - then the escalating class imbalance means that relying too much on distance will perform a lot better than ignoring it. The Discussion also suggests that predicting how distal enhancers are linked to target genes may be required for models to learn long-range regulatory interactions. I noted that Supplementary Figure 12 contains one approach ("Enformer-ABC") that may be relevant to this discussion. While I recognize that evaluating the Enformer-ABC model is outside of the scope of the current study, it may be of interest to mention the possibilities of this approach in the relevant section of the Discussion.*

*>We agree with the reviewer that this may be of interest to the field and that our preliminary step in this direction may remain unnoticed. We have now added the following text to our discussion of implications for model development:*

*>>Until a sequence-based model is developed which can correctly account for the impact of enhancers at any distance, we note that Enformer could also be used to extend more conventional approaches. Specifically, the activity-by-contact (ABC) model, which combines epigenetic profiles with Hi-C contact data to predict enhancer effects, has recently been demonstrated to be a useful method to study enhancers (Fulco et al. 2019; Hecker et al. 2023). As we started to explore (Additional file 1: Fig. S12), Enformer could be used to predict the activity component, which would allow the ABC model to be more easily applied to the the contact map.study of enhancer variants, under the assumption that these variants do not impact*

Reviewer #2:

In this work, Karollus et al. undertake a deep investigation of state-of-the-art models for predicting expression from sequence. Such an investigation is very timely as several deep learning approaches have received wide attention in recent years and each claims to be highly accurate in predicting gene expression, but the concept of "predicting expression" is rather nuanced and multi-faceted and it is not quite clear what exactly these methods are good at and what their weaknesses might be. The authors test the leading methods for this task in a wide variety of settings and make clear-cut inferences. The evaluation procedures are designed with care and a lot of effort. The work is impressive in its examination of a number of different sequence- expression datasets and reaching coherent conclusions from a harmonized modeling of these diverse sources. The analysis reported in Figure 3, showing the interplay of distance from TSS, cis-regulatory signal and model performance, is very nicely done and presented. I believe this in itself will be of great value to readers. The analysis reported in Figure 4 presents useful insights into the capabilities of the Enformer model. The in silico experiment suggesting that disruption of a CAT-box may be responsible for a particular observation related to placement of the TATA box is appealing. Through Figure 5, the authors offer convincing evidence that even the best sequence models today are poor at predicting regulatory impact of distal enhancers and distal eQTLs. This is a very important message for the community, and in my opinion the demonstration is sound and clear.

All in all, this is an excellent and highly significant work. I have only a couple of minor suggestions for improvement of the manuscript.

*>We thank the reviewer for their kind words.*

Minor comments:

The first subsection of Results reads a little abrupt and hurried as it stands. It seems to assume that the reader has already read the Methods or is familiar with how the general paradigm of training and testing these models works.

*>We were not sure on how to address this point. We had received good feedback from experimentalists at a conference about figure 1, which they found helpful to understand the study. We do not think that explaining the machine learning paradigm of supervised-learning and train and test data random splitting would be helpful here because we are instead reconstructing in-silico sequence that had generally been experimentally produced. To make the start of the Results section less abrupt, we have now added a short paragraph (see response to the last point). We also now refer explicitly to the Methods section. We hope this addresses the concern of this reviewer. We are happy to further improve the text with more specific prompts.*

The strategy of reporting accuracy on human tissues by applying a separately trained regressor (Figure 2B) appears somewhat misleading about predictive ability of Enformer, which only predicts the ENCODE CAGE tracks and the reported accuracy (and trends therein) may also reflect performance of the regressor (or some interaction between the Enformer predictions and the regressor). The authors should address this potential doubt that may arise in the reader's mind.

*>The reviewer is correct to point out that Enformer does not directly predict for human tissues. We agree that the section as it was written was insufficiently clear on this point and we thank the reviewer for making us aware of this. We added the following text to clarify:*

*>>Note that Enformer (and the other models we consider) were not trained on these datasets specifically and do not directly give predictions for many human tissues. To match CAGE tracks to tissues and stages of development in a simple, yet data-driven, way, we fitted a ridge regression. We held out the Enformer test set genes from this fitting, so as to prevent any data leakage. We evaluate the performance only on these test set genes. Therefore, in this section, when we refer to the predictions of a model, we mean the predictions of the model matched to the respective tissue/stage of development using the ridge regression.*

*>We agree with the reviewer that the choice of matching procedure will impact the overall performance figures we report. We consider the linear regression approach we chose as the simplest way to get Enformer to predict for human tissues, since it requires few additional parameters and no knowledge of human cellular anatomy. More sophisticated nonlinear regressors might yield better results at the cost of additional engineering effort, but as our goal is to mostly analyze the predictive ability of Enformer itself, we considered a simple approach to be most representative.*

*>Because the model is linear, we consider it somewhat unlikely that complex interactions between the Enformer predictions and the regressor could arise. Nevertheless, this is a valid concern we had not considered. To get a point of comparison, we therefore also matched CAGE tracks to tissues using a manual, model-free, procedure, by looking for relevant keywords in the descriptions of the respective tracks. Unsurprisingly, the results are worse than what we see with the linear model, although an expert in human cellular anatomy may be able to improve upon this. The between-tissue prediction suffers most of all, as the individual errors which arise from a tissue being matched badly compound once we study inter-tissue variation. However, the main trends we identify and discuss still hold: Enformer is still clearly the best model, it still predicts better for early development than later life stages and there is a similar (rho = 0.75) ranking of tissues in terms of how well they are predicted by Enformer.*

*>We added the following section to the text to summarize these results:*

*>>Linear regression is a simple data-driven approach to match CAGE tracks to tissues which requires no prior knowledge of human anatomy or sophisticated machine learning techniques. However, in some scenarios a data-driven matching may be impossible, e.g. if we wanted to predict for a tissue where no expression data is available. In this case, one would need to manually select tracks based on prior knowledge. We repeated the analyses of this section using a manual matching based on keywords (Additional file 2). Unsurprisingly, the resulting performance is worse than what can be achieved using our data-driven matching, but the conclusions are similar.*

The concept of "reproducing an experiment in silico" should be gently introduced at the beginning of the Results section; it was not clear what exactly was meant by this phrase, though it did become clear during the course of reading the results section.

*>We added the following paragraph to the beginning of this section:*

*>>In this study we conduct in-silico reproductions of experiments using sequence- based models of gene expression. By this we mean that we adapt the input of the model in such a way as to simulate, as faithfully as possible, the conditions of the experiment. We then evaluate to what extent the predictions of the model agree, qualitatively and quantitatively, with the findings of the experiment.*

*We hope that this helps to elucidate what we generally mean by in-silico reproduction.*

Line 130: Typo: should be lowercase 'w' in 'We'.

*>We thank the reviewer for making us aware of this typo.*
